# Diagnostic Performance of Neurofilaments in Chinese Patients With Amyotrophic Lateral Sclerosis: A Prospective Study

**DOI:** 10.3389/fneur.2018.00726

**Published:** 2018-08-28

**Authors:** Da-Wei Li, Haitao Ren, Andreas Jeromin, Mingsheng Liu, Dongshao Shen, Hongfei Tai, Qingyun Ding, Xiaoguang Li, Liying Cui

**Affiliations:** ^1^Department of Neurology, Xuanwu Hospital, Capital Medical University, Beijing, China; ^2^Department of Neurology, Peking Union Medical College Hospital, Chinese Academy of Medical Sciences, Beijing, China; ^3^Iron Horse Diagnostics, Inc., Scottsdale, AZ, United States; ^4^Neurosciences Center, Chinese Academy of Medical Sciences, Beijing, China

**Keywords:** amyotrophic lateral sclerosis, neurofilament protein, tau protein, diagnostic test, CSF biomarkers

## Abstract

Several studies have attempted to reduce diagnostic delay and identify biomarkers for drug development in amyotrophic lateral sclerosis (ALS). In this study, we aimed to evaluate the diagnostic accuracy for ALS of cerebrospinal fluid (CSF) neurofilament (Nf), Tau protein, and inflammatory factors such as interleukin (IL)-2, IL-6, IL-10, IL-15, and granulocyte-macrophage colony-stimulating factor (GMCSF) in Chinese patients. Our prospective study measured the concentration of phosphorylated Nf heavy chain (pNfH), Nf light chain (NfL), Tau, pTau, and inflammatory factors in the CSF of 85 patients. Detailed clinical data and laboratory, neuroimaging, and neurophysiological findings were recorded. The concentrations of pNfH and NfL were higher in the ALS group than in the control group. At the 1104 pg/mL pNfH cutoff, the specificity was 68.8%, the sensitivity 100%, and the area under the curve (AUC) 0.907. At the 1,139 pg/mL NfL cutoff, the specificity was 56.3%, the sensitivity 96.2%, and the AUC 0.775. There was no significant difference in the concentrations of Tau, pTau, IL-2, IL-6, IL-10, IL-15, and GMCSF between the ALS and control groups (*p* > 0.05). In the ALS group, the concentration of pNfH in the CSF was correlated with disease duration (*r* = −0.475, *p* < 0.001). This is the first prospective study to confirm the diagnostic value of Nf for ALS in Chinese patients.

## Introduction

Amyotrophic lateral sclerosis (ALS) is a fatal and progressive neurodegenerative disease characterized by degeneration of upper and lower motor neurons (1).

Diagnosis of ALS can be challenging at the early disease stage; the time from symptom presentation to diagnosis is approximately 12 months in most patients, by which time the patient may be beyond the window of therapeutic opportunity (2, 3). Validated biomarkers to detect ALS at the early disease stage are urgently needed to shorten diagnostic delay and facilitate differential diagnosis (3, 4).

Currently, the phosphorylated neurofilament heavy chain (pNfH) and neurofilament light chain (NfL) are the most promising candidate biomarkers for ALS (5). Several studies and meta-analyses have provided evidence of significantly increased concentrations of pNfH and NfL in the cerebrospinal fluid (CSF) of patients with ALS compared with those in the CSF of healthy and disease controls (5–14). However, many studies that have measured pNfH and NfL were retrospective. Moreover, the diagnostic utility of pNfH and NfL should be evaluated in the Chinese population because there is some heterogeneity in the age of disease onset, genetic basis, and median survival time of ALS between Chinese and Western patients (14, 15).

In addition, other putative CSF biomarkers have been reported in patients with ALS, such as phosphorylated tau (pTau), Tau, and the neuroinflammatory factors, interleukin (IL)-2, IL-6, IL-10, IL-15, and granulocyte-macrophage colony-stimulating factor (GMCSF) (16–19). However, many studies that assessed the diagnostic performance of CSF pTau, Tau, IL-2, IL-6, IL-10, IL-15, and GMCSF in patients with ALS have reported inconsistent results.

The objectives of this study were to evaluate the diagnostic performance of pNfH, NfL, pTau, Tau IL-2, IL-6, IL-10, IL-15, and GMCSF in Chinese patients with ALS, at the early diagnostic stage, when the neurologist is in doubt of the diagnosis of ALS.

## Materials and methods

### Participants and clinical characterization

We performed a prospective analysis of consecutively acquired data from patients who attended our clinic for ALS at the Peking Union Medical College Hospital between May 2015 and November 2016. We included consecutive patients who fulfilled the revised El Escorial criteria (rEEC) for suspected ALS, and patients with non-ALS neurological disorders, as controls (Supplemental Data) at initial investigation (20). We excluded any patients from which we did not obtain a CSF sample in our laboratory. This study was conducted in accordance with the Declaration of Helsinki, and the protocol was approved by the Ethics Committee of Peking Union Medical College Hospital. All participants provided written informed consent (or gave verbal permission for their legal next of kin to sign on their behalf).

Demographic and clinical characteristics of patients were obtained directly from patients or from medical records. These included age, disease duration, and phenotype of ALS. Patient functional status was determined using the ALS Functional Rating Score-Revised (ALSFRS-R) (21). Disease duration was defined as the time between onset of bulbar symptoms or first weakness and lumbar puncture.

### CSF collection and analysis

CSF samples were collected by lumbar puncture, centrifuged, aliquoted, and stored at −80°C within 2 h of collection (22, 23). The concentration of markers in the CSF samples was measured in December 2016. Investigators were blinded to the clinical data. Commercially available enzyme-linked immunosorbent assay (ELISA) kits were used to measure pNfH, pTau, and Tau (EUROIMMUN, Lübeck, Germany); NfL (Uman Diagnostics, Umea, Sweden), IL-2, IL-15, and GMCSF (R&D Systems, Minneapolis, MI, USA); and IL-6 and IL-10 (Invitrogen, Carlsbad, CA, USA) concentrations, according to the manufacturers' instructions.

### Reference standard

The reference standard that was used to confirm the diagnosis of ALS met the rEEC (clinically probably ALS or definitely ALS) and a disease progression during follow-up for at least 6 months (24).

### Statistical analysis

Statistical analyses were performed using SPSS 17.0 (IBM Corporation, Chicago, IL, USA) and MedCalc 17.2 (MedCalc, Mariakerke, Belgium). Data were reported as median (interquartile range) or mean ± standard deviation. Most data showed a non-normal distribution; therefore, intergroup differences were assessed for significance using the 2-tailed unpaired Mann-Whitney *U*-test. The diagnostic accuracy of NfL and pNfH was assessed using receiver operating characteristic (ROC) curves. An optimal cutoff was calculated using Youden's index (25). We used MedCalc to calculate the sensitivity, specificity, and the area under the curve (AUC) for the optimal NfL and pNfH cutoffs and predictive values with corresponding 95% confidence intervals (CI). Spearman's rank correlation coefficient was calculated to analyze the non-normally distributed data. *p* < 0.05 was considered statistically significant.

## Results

### Demographic and clinical characteristics of the subjects

We recruited 85 patients in a prospective study design, including 53 patients with ALS, seven patients with ALS mimics disease, and 25 patients with non-ALS neurological disorders, as controls. Due to the small number of patients with ALS mimics disease, we combined the ALS mimics and non-ALS neurological groups (*n* = 32). The demographic and clinical characteristics of the subjects are listed in Table 1. There were no differences in age, sex, and disease duration between the ALS and control groups. Forty patients (75.5%) had the spinal-onset form of ALS.

**Table 1:** Clinical characteristics of the participants at the time of the lumbar puncture.

	**ALS**	**Control**
*n*	53	32
Male	62.3% (33/53)	68.8% (22/32)
Female	37.7% (20/53)	31.2% (10/32)
Age (years)	50.68 ± 12.66	50.53 ± 10.65
Disease duration (months)	13.87 ± 9.9	14.8 ± 13.21
Age of onset (years)	49.04 ± 12.74	
**SITE OF ONSET**		
spinal	75.5% (40/53)	
bulbar	24.5% (13/53)	
ALSFRS-R	38.98 ± 5.23	

*ALSFRS-R, ALS Functional Rating Score-Revised*.

### Group differences in CSF biomarkers

#### CSF pNfH

**Figure 1:**
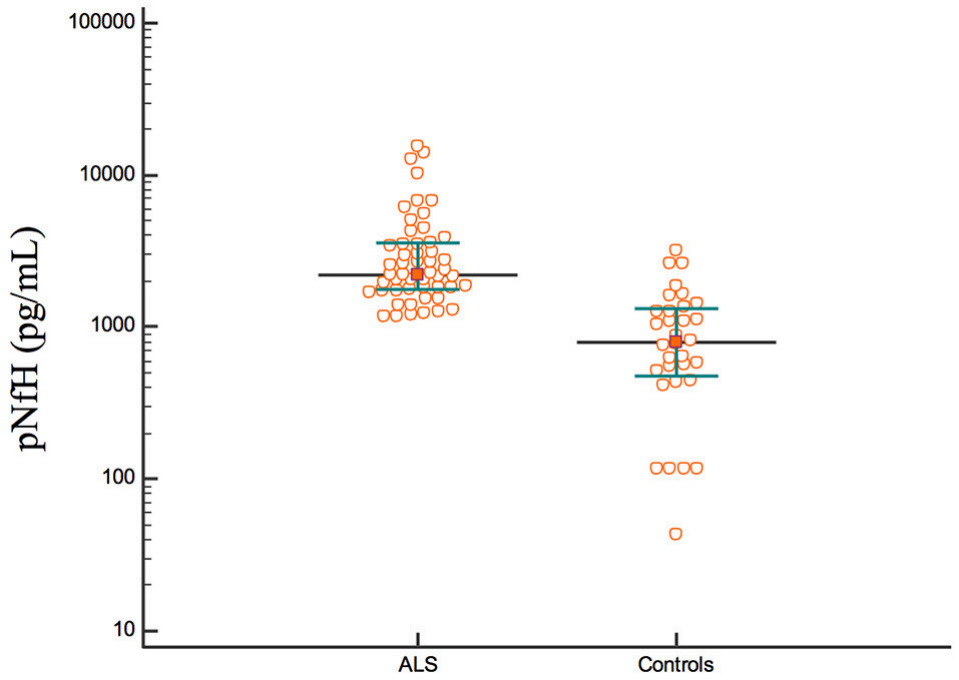
The concentrations of pNfH in the ALS and control group.

The concentration of pNfH was higher in the ALS group than in the control group [ALS: median, 2189 pg/mL; interquartile range (IQR), 1,757–3,560 pg/mL; control: median, 792 pg/mL; IQR, 465–1330 pg/mL; *p* < 0.001; Figure 1].

#### CSF NfL

**Figure 2:**
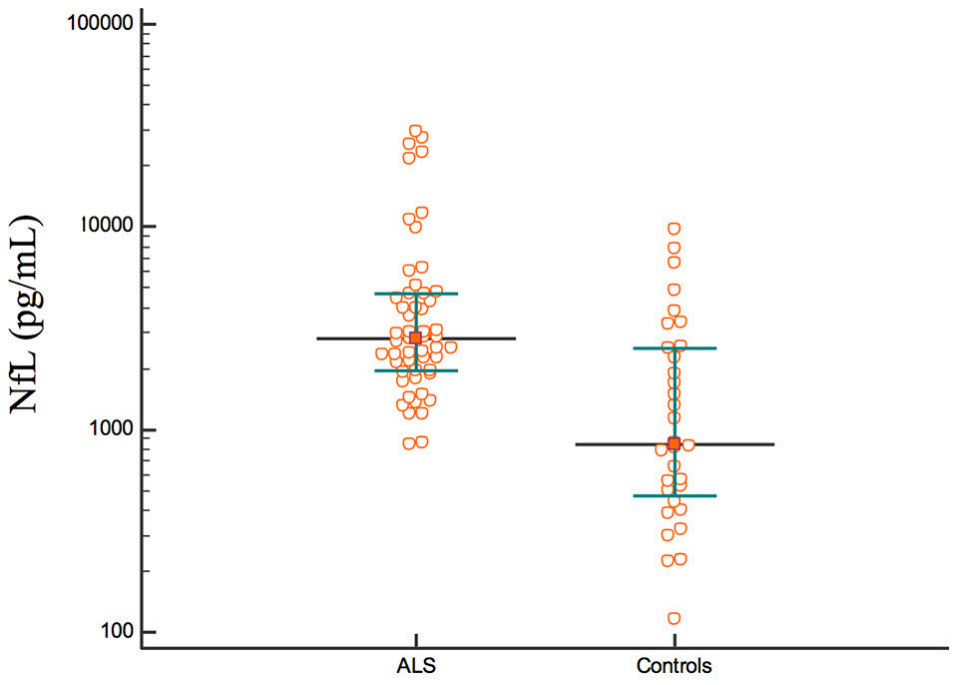
The concentrations of NfL in the ALS and control group.

The concentration of NfL was higher in the ALS group than in the control group (ASL: median, 2,823 pg/mL, IQR, 1,933–4,658 pg/mL; control: median, 845 pg/mL, IQR, 455–2,552 pg/mL; *p* < 0.001; Figure 2).

#### CSF Tau, pTau, IL-2, IL-6, IL-10, IL-15, and GMCSF

There was no significant difference in the concentrations of CSF Tau, pTau, IL-2, IL-6, IL-10, IL-15, and GMCSF between the ALS and control groups (*p* > 0.05).

#### Applying cutoffs for pNfH and NfL in all ALS patients

A pNfH concentration higher than 1104 pg/mL yielded the optimal discrimination between ALS and control patients, with a sensitivity and specificity of 100% (95% CI, 93.3–100) and 68.8% (95% CI, 50–83.9), respectively.

The best diagnostic performance was gained at a cutoff of 1,139 pg/mL NfL, with a sensitivity and specificity of 96.2% (95% CI, 87–99.5) and 56.3% (95% CI, 37.7–73.6), respectively.

**Figure 3:**
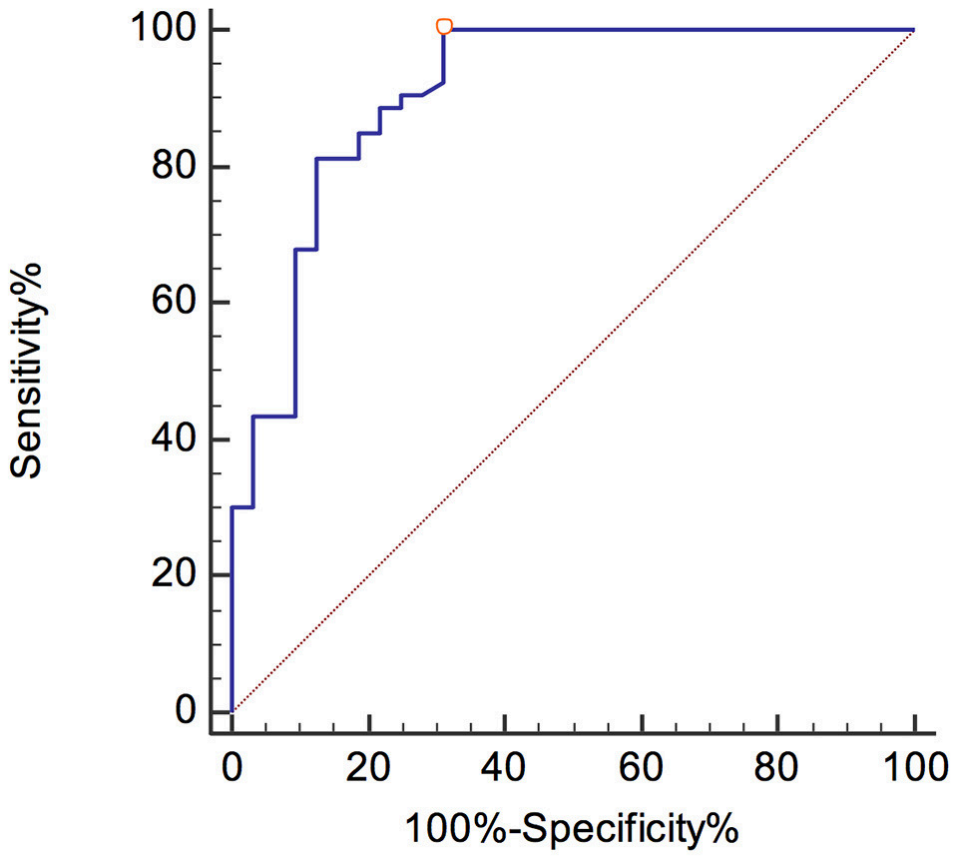
ROC curve for pNfH levels in the ALS and control group.

**Figure 4:**
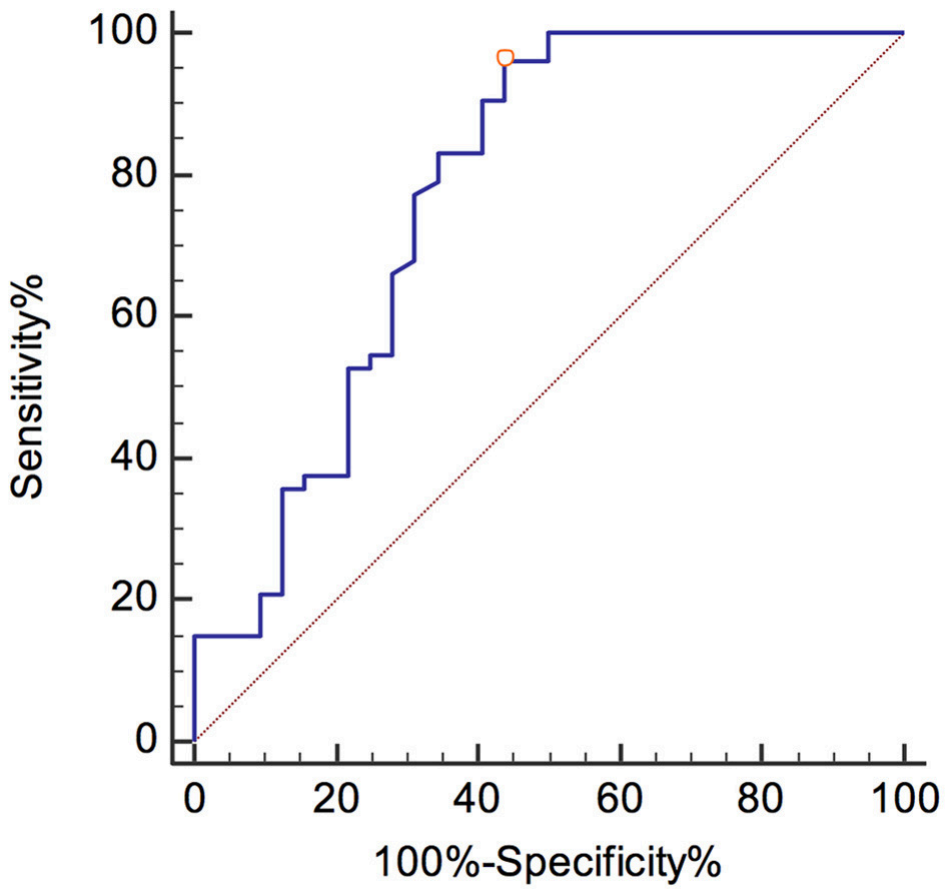
ROC curve for NfL levels in the ALS and control group.

The ROC curves are shown in Figures 3, 4. The corresponding AUCs for pNfH and NfL were 0.907 ± 0.036 (95% CI, 0.825–0.959; Figure 3) and 0.775 ± 0.059 (95% CI, 0.671–0.858; Figure 4), respectively.

#### Applying cutoffs for pNfH and NfL in ALS patients at the early disease stage (disease duration not exceed 12 months)

Thirty-five ALS patients were analyzed. A pNfH concentration higher than 1,662 pg/mL yielded the optimal discrimination between ALS and control patients, with a sensitivity and specificity of 82.9% (95% CI, 66.4–93.4) and 87.5% (95% CI, 71–96.5), respectively.

The best diagnostic performance was gained at a cutoff of 1,307 pg/mL NfL, with a sensitivity and specificity of 91.4% (95% CI, 76.9–98.2) and 59.4% (95% CI, 40.6–76.3), respectively.

The corresponding AUCs for pNfH and NfL were 0.918 ± 0.033 (95% CI, 0.825–0.971) and 0.772 ± 0.06 (95% CI, 0.654–0. 866), respectively.

#### Correlation between CSF compounds and clinical parameters of ALS

**Figure 5:**
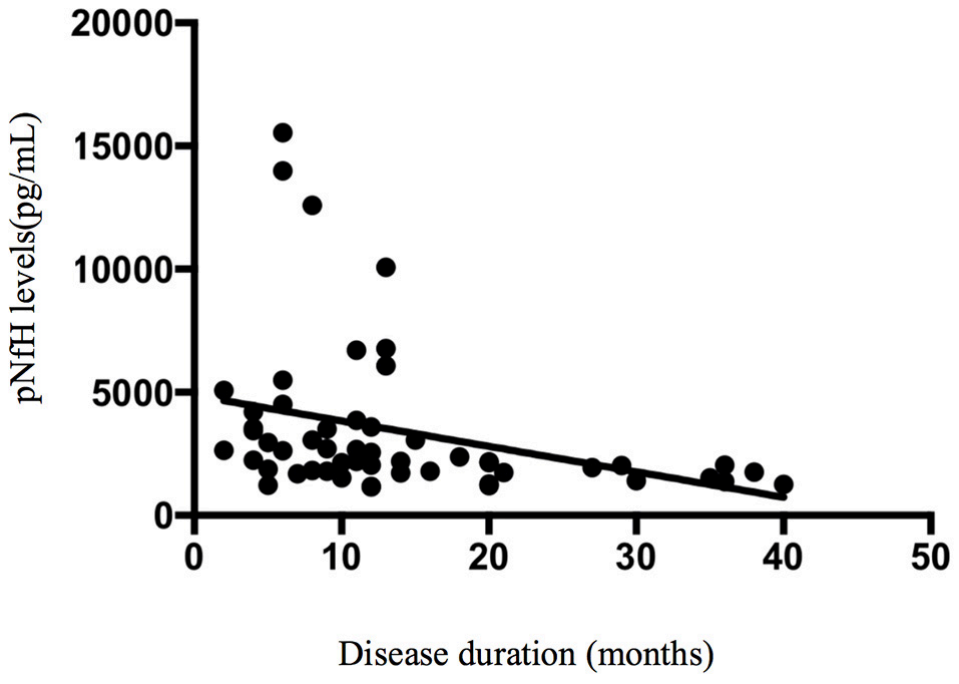
An inverse correlation between CSF pNfH and disease duration.

**Figure 6:**
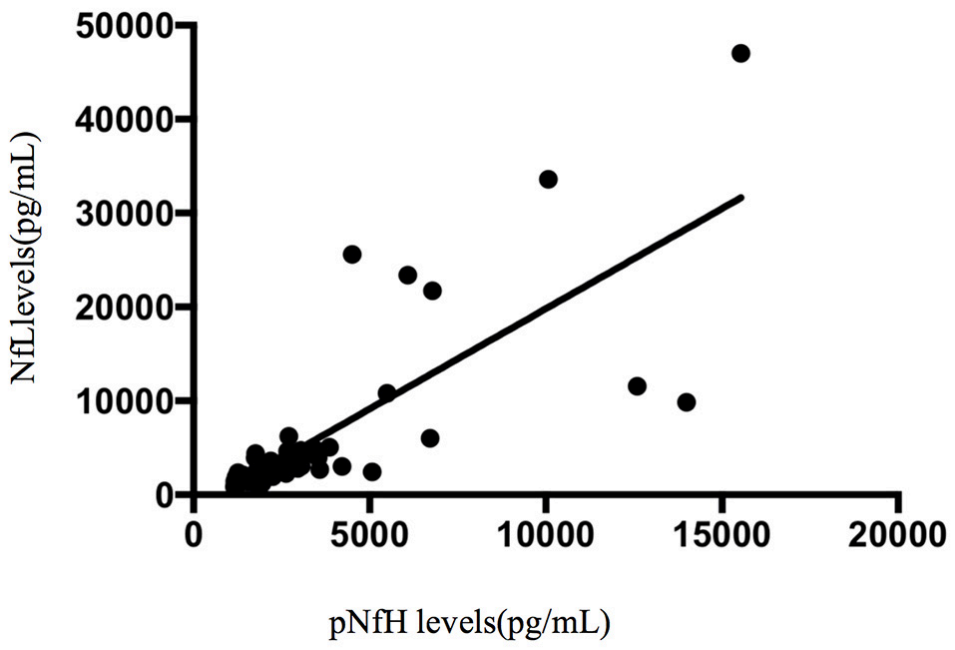
A positive correlation between CSF pNfH and NfL.

Spearman's rank correlation coefficients showed that, in the ALS group, the concentration of pNfH in the CSF was correlated with disease duration (*r* = −0.475, *p* < 0.001; Figure 5). In addition, the concentration of pNfH was correlated with the concentration of NfL (*r* = 0.8, *p* < 0.001; Figure 6). There were no significant correlations between the concentration of pNfH and age or ALSFRS-R. In addition, there were no significant correlations between the concentration of CSF NfL, Tau, pTau, and age, disease duration, and ALSFRS-R. The subgroups of patients with bulbar or spinal onset ALS had similar concentrations of pNfH, NfL, Tau, and pTau.

## Discussion

To the best of our knowledge, this is the largest prospective study to have assessed and confirmed pNfH and NfL as potential biomarkers for ALS in Chinese patients. Furthermore, we did not observe any significant differences in the concentrations of CSF Tau, pTau, IL-2, IL-6, IL-10, IL-15, and GMCSF between the ALS and control groups.

Several studies and meta-analyses have shown that the concentrations of CSF pNfH and NfL are significantly increased in patients with ALS (5, 7, 8, 14, 26). The most recent meta-analysis reported a diagnostic sensitivity and specificity of pNfH 85% (95% CI, 80–88%) and 85% (95% CI, 77–90%), respectively, and AUC value of 0.91 (95% CI, 0.88–0.93) (14). We found a similar diagnostic sensitivity, specificity, and AUC for CSF pNfH levels in this study; however, the optimal cutoff for pNfH (>1104 pg/mL) in our study was higher than in most other studies (437–1,200 pg/mL). We speculate that this difference may be related to research design, control-group choice, disease-progression rate, and disease duration (27, 28). A statistically significant inverse correlation was found between CSF pNfH level and disease duration, suggesting that the concentration of pNfH may be higher early in the disease course. Our study included patients with a shorter disease duration than previous studies.

NfL is considered another diagnostic marker of ALS. The previous meta-analysis reported an NfL diagnostic sensitivity and specificity of 81% (95% CI, 72–88%) and 85% (95% CI, 76–91%), respectively, and AUC 0.90 (95% CI, 0.87–0.92) (14). The NfL diagnostic sensitivity, specificity, and AUC in our study were lower than they were in this meta-analysis. In addition, to the reasons mentioned above, we consider that this may be the related to NfL stability or severity of disease (28, 29). NfL levels are higher in patients with more severe disease presentation.

NfL and pNfH levels have excellent diagnostic sensitivity and specificity to diagnose ALS, even in the early stage of the disease which disease duration did not exceed 12 months. However, pNfH had better AUC, sensitivity, and specificity than NfL in our study design. Hence, our study proposes pNfH as the best diagnostic biomarker for ALS. Nevertheless, the results showed that care should be taken when applying a reported cutoff to a given ALS cohort. It is advised that neuromuscular reference centers should validate the performance/cutoff of the markers based on the findings of their own patients with ALS.

Tau protein is a marker of cytoskeletal axonal degeneration. Our study did not find any significant differences in Tau and pTau concentrations between the ALS and control groups. The heterogeneity of Tau protein diagnostic performance for ALS indicates that the result from our study needs to be considered (6, 30). We argue that different ELISA methods were used among studies, which may explain the differences.

Previous studies have reported higher concentrations of IL-2, IL-6, IL-8, IL-15, and GMCSF and a lower concentration of IL-10 among ALS groups (18, 19, 31); however, this study found no statistically significant difference pertaining to these factors. Our results do not negate the involvement of central nervous system inflammatory mechanisms in the onset of ALS (32). We hypothesize that our results may be attributable to changes in inflammatory mechanisms in the control group.

This study had some limitations. We recruited a small number of patients and did not include a validation cohort to further verify the results; however, we argue that our data reflect the characteristics of pNfH and NfL in Chinese patients with ALS. Moreover, numerous nervous-system inflammatory diseases were present in the control group and the levels of cytokines (e.g., IL-2, IL-6, IL-10) in the CSF were correlated with numerous nervous-system inflammatory diseases, which led to the absence of significant differences between the ALS and non-ALS groups regarding these factors.

In conclusion, this study confirmed the role of pNfH and NfL as diagnostic biomarkers for ALS in Chinese patients. Furthermore, pNfH has potentially useful diagnostic sensitivity and specificity. pNFH in the CSF and serum should be clinically validated further by longitudinal and multi-center studies.

## Author contributions

D-WL and LC designed and developed the study protocol. D-WL, DS, HT, and QD performed the literature review and statistical analyses. D-WL and HR performed the measurements and wrote the first draft of the manuscript. AJ, ML, XL, and LC reviewed and critiqued the manuscript.

### Conflict of interest statement

AJ was employed by the company Iron Horse Diagnostics.

The remaining authors declare that the research was conducted in the absence of any commercial or financial relationships that could be construed as a potential conflict of interest.
