# Supplementary material for: Diagnostic Performance of Neurofilaments in Chinese Patients With Amyotrophic Lateral Sclerosis: A Prospective Study
Source: Front Neurol. 2018 Aug 28;9:726. doi: 10.3389/fneur.2018.00726 (PMC6121092; doi:10.3389/fneur.2018.00726)
Supplement: Supplementary file 1 [file Data_Sheet_1.DOCX]

Supplemental data: non-ALS neurologic controls

The control group comprised 32 patients who were final diagnosed as following:

1 Cervical spondylotic myelopathy

2 Cervical spondylotic myelopathy

3 Thyroid Carcinoma

4 Cervical spondylotic myelopathy

5 Cervical spinal radiculopathy

6 Cervical radiculopathy

7 Hemangioblastoma (C4)

8 Polyradiculoneuropathy

9 Polyradiculoneuropathy

10 Peripheral neuropathy

11 Peripheral neuropathy

12 Peripheral neuropathy

13 IgLON5 encephalopathy

14 Immune-mediated peripheral neuropathy

15 Hypoglossal nerve palsy, Epidural hyperplasia

16 Subacute combined degeneration of the spinal cord

17 Intracranial Hypertension

18 Viral encephalitis

19 Brain stem brain inflammation

20 Multiple system atrophy

21 Chronic inflammatory demyelinating polyradiculoneuropathy

22 Cerebellar ataxia

23 Cerebral pachymeningitis

24 Papilledema

25 Peripheral neuropathy

26 Cervical spondylotic myelopathy

27 Cortical thrombosis

28 Hepatic encephalomyeloopathy

29 Myasthenia gravis, lumbar disc herniation

30 Pseudomonas aeruginosa infection, agammaglobulinemia

31 IgLON5 encephalopathy

32 IgLON5 encephalopathy

The first seven patients presented with ALS mimics.

We used clinical, neurophysiological, laboratory, and neuroimaging data to identify ALS mimic disorders:

1. Cervical compressive myelopathy: cervical spine MRI (significant cord compression with intrinsic spinal cord signal abnormality) + nerve conduction studies + electromyography + clinical signs (sensory symptoms and signs, Lhermitte' s symptom)

2. Hemangioblastoma：imaging + electromyography + surgery + pathology

3. Paraneoplastic syndrome: serum markers + imaging + electromyography + repetitive stimulation

4. Multifocal motor neuropathy (MMN): nerve conduction studies (multifocal nerve conduction block) + electromyography + clinical signs (absent upper motor neuron involvement) + high anti-GM1 IgM antibodies.
